# Identification of key sex-specific pathways and genes in the subcutaneous adipose tissue from pigs using WGCNA method

**DOI:** 10.1186/s12863-022-01054-w

**Published:** 2022-05-10

**Authors:** Huiyu Wang, Xiaoyi Wang, Mingli Li, Shuyan Wang, Qiang Chen, Shaoxiong Lu

**Affiliations:** 1 Faculty of Animal Science and Technology, Yunnan Agricultural University, No. 95 of Jinhei Road, Kunming, 650201 Yunnan China; 2 Faculty of Animal Science, Xichang University, Xichang, 615000 Sichuang China

**Keywords:** Sex, Pigs, Subcutaneous fat tissue, WGCNA, Key pathways and genes

## Abstract

**Background:**

Adipose tissues (ATs), including visceral ATs (VATs) and subcutaneous ATs (SATs), are crucial for maintaining energy and metabolic homeostasis. SATs have been found to be closely related to obesity and obesity-induced metabolic disease. Some studies have shown a significant association between subcutaneous fat metabolism and sexes. However, the molecular mechanisms for this association are still unclear. Here, using the pig as a model, we investigated the systematic association between the subcutaneous fat metabolism and sexes, and identified some key sex-specific pathways and genes in the SATs from pigs.

**Results:**

The results revealed that 134 differentially expressed genes (DEGs) were identified in female and male pigs from the obese group. A total of 17 coexpression modules were detected, of which six modules were significantly correlated with the sexes (*P* < 0.01). Among the significant modules, the greenyellow module (cor = 0.68, *P* < 9e-06) and green module (cor = 0.49, *P* < 0.003) were most significantly positively correlated with the male and female, respectively. Functional analysis showed that one GO term and four KEGG pathways were significantly enriched in the greenyellow module while six GO terms and six KEGG pathways were significantly enriched in the green module. Furthermore, a total of five and two key sex-specific genes were identified in the two modules, respectively. Two key sex-specific pathways (Ras-MAPK signaling pathway and type I interferon response) play an important role in the SATs of males and females, respectively.

**Conclusions:**

The present study identified some key sex-specific pathways and genes in the SATs from pigs, which provided some new insights into the molecular mechanism of being involved in fat formation and immunoregulation between pigs of different sexes. These findings may be beneficial to breeding in the pig industry and obesity treatment in medicine.

**Supplementary Information:**

The online version contains supplementary material available at 10.1186/s12863-022-01054-w.

## Background

It is well known that adipose tissue (AT) is a kind of central metabolic tissue of complex and highly metabolically activity, and participates in regulating systemic energy homeostasis [1]. AT has key roles in the pathogenesis of obesity and obesity-induced metabolic disease by secreting hormones, cytokines and adipokines involving the regulation of metabolism [2, 3]. The ATs located in the abdominal and thoracic cavities are called visceral ATs (VATs), which have been considered anatomically, functionally and metabolically significantly different from compartmental subcutaneous ATs (SATs) [4]. It has been found that SATs are closely related to obesity and obesity-induced metabolic disease [5]. Pigs (*Sus scrofa*) are important biomedical models for studying energy metabolism and human diseases, such as obesity, type II diabetes, and cardiovascular diseases because their body size and physiological/anatomical features are similar to those of humans [6]. And it offers the possibility of in-depth study of the transcription levels of SATs, but this is difficult in humans.

At present, most of the studies mainly focused on obesity study for SATs using pigs as a model and identified some important pathways and genes related to obesity [7–9]. Nevertheless, little attention was paid to the gender difference in obesity. In recent years, some studies have shown a significant association between subcutaneous fat metabolism and sexes [10–12]. Despite some progress, the molecular mechanisms of fat formation and metabolism in SATs involved in gender are still unclear. Especially, the coexpression relationship of sex-specific genes in SATs remains unknown.

Weighted Gene Coexpression Network Analysis (WGCNA) is a systematic biology method to describe the correlation patterns among genes across samples [13]. Compared with other methods, WGCNA focuses on the relationship between coexpression modules and phenotypes [14]. Using WGCNA can find the gene coexpression modules with higher reliability and biological significance, and identify “driver” genes in the modules [15]. Currently, WGCNA has become the most important way to study the coexpression relationships among genes and has been successfully applied in various research fields, such as complex diseases, including hepatocellular carcinoma [16], uveal melanoma [17], hyperlipidemia [18], and obesity [8, 19], and economic traits, including meat quality [20], hypoxic adaptation [21] and skin color [22], etc. Lim et al. identified functional modules and hub genes, which were related to a marbling trait in Hanwoo (Korean) cattle using WGCNA method. These hub genes were mainly involved in biological processes, which were correlated with fat or muscle formation [23]. Xing et al. found that four coexpression modules were significantly correlated with the backfat thickness in Songliao black and Landrace with high and low backfat using WGCNA method [24]. Besides, protein and protein interaction (PPI) networks are also viable tools to construct a gene coexpression network and understand cell functions and disease machinery [25]. Zhao et al. identified *ADIPOQ*, *PPARG*, *LIPE*, *CIDEC*, *PLIN1*, *CIDEA*, and *FABP4* as potential candidate genes affecting intramuscular fat (IMF) content in 28 purebred Duroc pigs by integrating the results from WGCNA and PPI methods [26].

In the present study, RNA-Seq data of abdominal subcutaneous adipose tissue (ASAT) of males and females (crossbred F2 of Duroc × Göttingen minipig) were retrieved from Gene Expression Omnibus (GEO) database and were systematically integrated and analyzed using WGCNA and PPI network analysis methods, with the aim to identify the significant modules closely related to the sexes, and further identify key sex-specific pathways and genes in the SATs of pigs. These findings may contribute to further understanding of the functions of porcine ATs and the mechanisms of regulating fat metabolism in SATs from pigs of different sexes, and provide some insights into the obesity treatment in medicine. Moreover, the identified key sex-specific genes may serve as potential biomarkers in pig breeding and potential targets in obesity treatment.

## Results

### Identification of differentially expressed genes (DEGs)

**Fig. 1:**
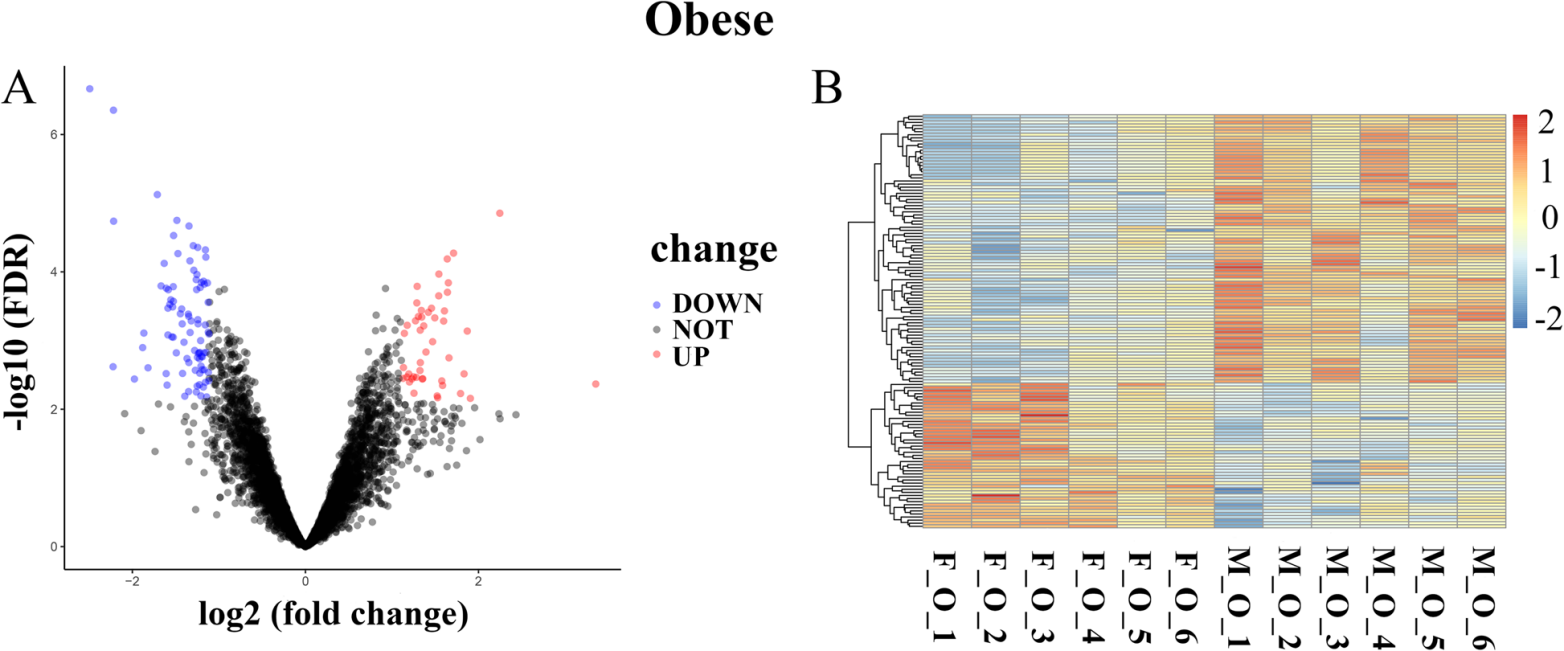
Differentially expressed genes (DEGs) analysis. **A** Volcano plot of all genes in the obese group. X-axis represented log2(fold change). Y-axis represented -log10(FDR). Blue spots represented down-regulated DEGs and red spots represented up-regulated DEGs. Black spots were not DEGs. DEGs (females compared with males). **B** Heatmap of all DEGs (females compared with males) in the obese group. X-axis represented samples. Y-axis represented genes. Blue represented down-regulated DEGs and red represented up-regulated DEGs. The color scale showed the expression values

By analyzing the transcriptome sequencing data of SAT of females and males in three groups (Lean, intermediate and obese groups) using the limma package, 134 DEGs (|log2FC|> 1, FDR < 0.1) were detected in the SAT of females and males in the obese group, of which 47 genes were significantly up-regulated and 87 genes were significantly down-regulated in females as compared with males (Fig. 1A, Table S3). However, no DEGs were identified in the lean and intermediate groups. The expression heatmap of all genes in the obese group was shown in Fig. 1B.

### WGCNA and the significant module identification

**Fig. 2:**
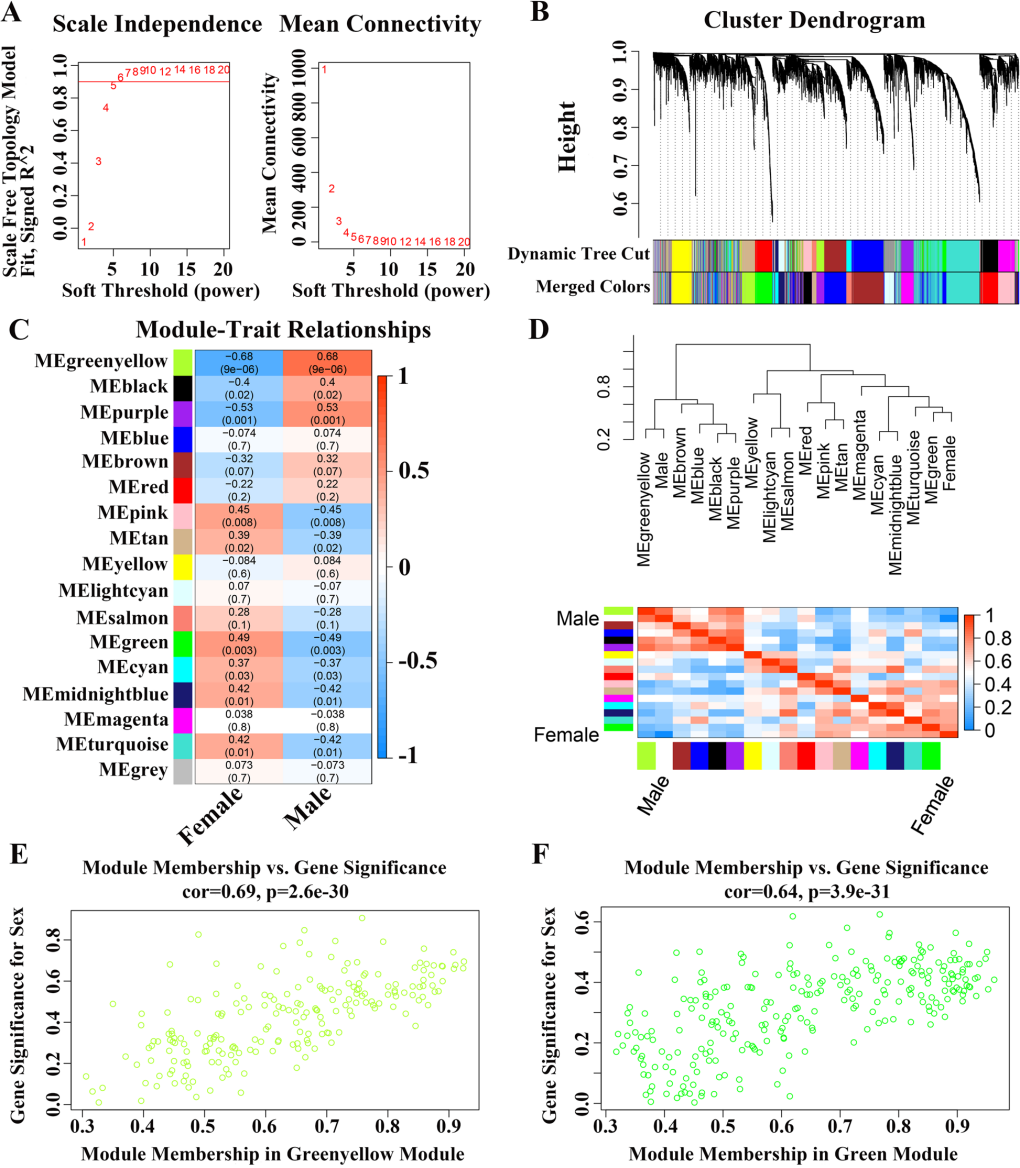
WGCNA. **A** Scale independence and mean connectivity of various soft-thresholding values (β). The left panel (**A**) displayed the influence of soft-thresholding power (X-axis) on the scale-free fit index (Y-axis). The right panel (**A**) showed the influence of soft-thresholding power (X-axis) on the mean connectivity (degree, Y-axis). **B** Cluster dendrogram of all filtered genes enriched based on the dissimilarity measure and the cluster module colors. **C** Matrix with Module-Trait Relationships (MTRs) and corresponding *P*-values between the detected modules on the y-axis and sexes (female and male) on the x-axis. **D** Heatmap of the adjacencies of modules. Red represented positive correlation and blue represented negative correlation. The male group clustered with the greenyellow module, and the female group clustered with the green module. Association between the module membership and gene significance within the greenyellow module (**E**) and the green module (**F**). WGCNA, weighted gene co-expression network analysis

The expression matrix containing 5000 genes was used to reconstruct the gene coexpression network by the WGCNA method. A Pearson correlation matrix among genes was converted into a strengthened adjacency matrix by power β = 5 based on the scale-free topology criterion with *R*^2^ = 0.9 (Fig. 2A). The topological overlap measure (TOM) of each gene pair was calculated. Seventeen gene coexpression modules were identified by an average linkage hierarchical clustering according to the TOM-based dissimilarity (1-TOM) (Fig. 2B). There were large differences in the number of genes among the modules. The lightcyan module with the minimum number contained 137 transcripts, while the turquoise module with the maximum number contained 855 transcripts (Table S2).

Correlation analysis between module eigengene (ME) and the sexes showed that six modules were significantly correlated with the sexes (*P* < 0.01). The modules of significantly positively correlated with the male were the greenyellow module (cor = 0.68 and *P* = 9e-06) and the purple module (cor = 0.53 and *P* = 0.001). The modules of significantly positively correlated with the female were the green module (cor = 0.49 and *P* = 0.003), the pink module (cor = 0.45 and *P* = 0.008), the midnightblue module (cor = 0.42 and *P* = 0.01), and the turquoise module (cor = 0.42 and *P* = 0.01) (Fig. 2C). The eigengene adjacency heatmap depicting the cluster relation of the identified modules and sexes was shown in Fig. 2D. It was found that the greenyellow module and the green module clustered with the male group and the female group, respectively. As above, the greenyellow module was most significantly positively correlated with the male, while the green module was most significantly positively correlated with the female. Furthermore, the correlation of module membership (MM) and gene significance (GS) in the greenyellow module (cor = 0.69 and *P* < 2.6e-30, Fig. 2E) and the green module (cor = 0.64 and *P* < 3.9e-31, Fig. 2F) indicated that the two modules possessed the top two significant correlations across all modules. Thus, the greenyellow module and the green module were selected for further analyses.

### Functional enrichment analysis and key genes identification for the greenyellow and green modules

GO and KEGG enrichment analyses were performed on all genes in the greenyellow and green modules using the Database for Annotation, Visualization and Integrated Discovery (DAVID). In the greenyellow module, GO enrichment results showed that one biological process (Activation of MAPK activity) was significantly enriched (*P* < 0.05). KEGG enrichment analysis showed that four KEGG pathways were significantly enriched (*P* < 0.05), including Ras signaling pathway, MAPK signaling pathway, Pathways in cancer and Melanoma. The significant enrichment terms were shown in Table 1. In the green module, GO enrichment results showed that four biological processes (Immune response, Chemokine-mediated signaling pathway, Lymphocyte chemotaxis and Cell chemotaxis) and two molecular functions (Chemokine activity and Double-stranded RNA binding) were significantly enriched (*P* < 0.05). KEGG enrichment analysis showed that six KEGG pathways were significantly enriched (*P* < 0.05), containing Cytosolic DNA-sensing pathway, Herpes simplex infection, Cytokine-cytokine receptor interaction, Chemokine signaling pathway, Measles and Toll-like receptor signaling pathway. The significant enrichment terms were shown in Table 2.

**Table 1:** The results of functional enrichment analysis for the greenyellow module using DAVID tool

ID	KEGG/GO terms	Gene symbols	*P*-value	Count
**KEGG**				
ssc04014	Ras signaling pathway	*IGF1*, *FGF1*, *FGF10*, *EGFR*, *LOC100522721*, *PLA1A*, *FOXO4*	0.009318916	7
ssc05200	Pathways in cancer	*IGF1*, *FGF1*, *FGF10*, *EGFR*, *LOC100522721*, *PLCB4*, *MMP2*, *TCF7L2*, *FZD5*	0.013129853	9
ssc04010	MAPK signaling pathway	*FGF1*, *FGF10*, *LOC100522721*, *EGFR*, *CACNA1G*, *GADD45G*, *LOC100620270*	0.014998697	7
ssc05218	Melanoma	*IGF1*, *FGF1*, *FGF10*, *EGFR*	0.018487192	4
**Biological process**				
GO:0,000,187	Activation of MAPK activity	*IGF1*, *FGF1*, *FGF10*, *C1QTNF2*	0.004864629	4

**Table 2:** The results of functional enrichment analysis for the green module using DAVID tool

ID	KEGG/GO terms	Gene symbols	*P*-value	Count
**KEGG**				
ssc04623	Cytosolic DNA-sensing pathway	*CXCL10*, *CCL5*, *ZBP1*, *DDX58*, *CCL4*	7.62E-04	5
ssc05168	Herpes simplex infection	*CCL5*, *LOC100157336*, *DDX58*, *TAP2*, *OAS2*, *OAS1 IFIT1*	0.001407528	7
ssc04060	Cytokine-cytokine receptor interaction	*CX3CL1*, *CXCL10*, *CCL5*, *CXCL9*, *CCL4*, *CXCL16*, *IL2RB*	0.002333772	7
ssc04062	Chemokine signaling pathway	*CX3CL1*, *CXCL10*, *CCL5*, *CXCL9*, *CCL4*, *CXCL16*	0.006306876	6
ssc05162	Measles	*DDX58*, *OAS2*, *MX1*, *OAS1*, *IL2RB*	0.012380535	5
ssc04620	Toll-like receptor signaling pathway	*CXCL10*, *CCL5*, *CXCL9*, *CCL4*	0.031557568	4
**Biological process**				
GO:0,006,955	Immune response	*CXCL10*, *CD244*, *CCL5*, *LOC100513601*, *CTSW*, *OAS2*, *OAS1*, *CXCL9*, *CCL4*	1.41E-05	9
GO:0,070,098	Chemokine-mediated signaling pathway	*CXCL10*, *CCL5*, *CXCL9*, *CCL4*	0.001153545	4
GO:0,048,247	Lymphocyte chemotaxis	*CCL5*, *CCL4*, *CXCL16*	0.005016185	3
GO:0,060,326	Cell chemotaxis	*CXCL10*, *CCL5*, *CCL4*	0.035772607	3
**Molecular function**				
GO:0,008,009	Chemokine activity	*CXCL10*, *CCL5*, *CXCL9*, *CCL4*, *CXCL16*	4.71E-05	5
GO:0,003,725	Double-stranded RNA binding	*DDX58*, *DHX58*, *OAS2*, *OAS1*	0.001718079	4

**Fig. 3:**
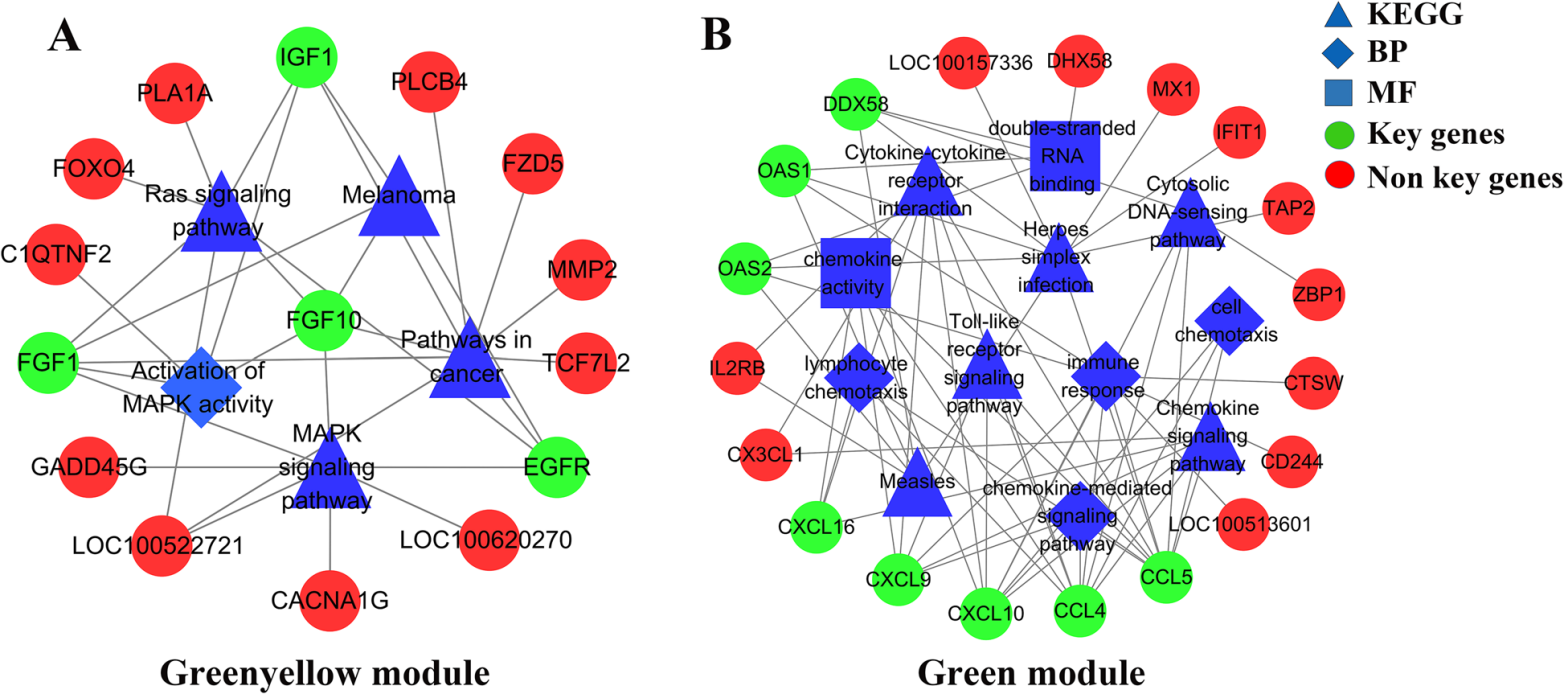
Pathway-gene interactive networks for the greenyellow and green modules. **A** Four KEGG pathways, one GO term and 14 genes were used to construct a pathway-gene interactive network for the greenyellow module. **B** Six KEGG pathways, six GO terms and 19 genes were used to construct a pathway-gene interactive network for the green module. Blue triangles represented KEGG pathway terms. Blue diamonds represented BP terms, and blue squares represented MF terms. Circles represented genes. Green circles represented key genes and red circles represented non key genes

In this study, the key genes were identified according to the criterion that the gene was at least involved in four KEGG/GO terms. So, four key genes (*FGF10*, *FGF1*, *EGFR* and *IGF1*) in the greenyellow module were identified (Fig. 3A). Among the four genes, *FGF10* and *IGF1* were significantly down-regulated in the obese group, while *FGF1* was significantly up-regulated in the obese group (Table S3**)**. In the green module, eight genes (*DDX58*, *OAS2*, *OAS1*, *CXCL9*, *CXCL10*, *CXCL16*, *CCL4* and *CCL5*) were selected as key genes (Fig. 3B). Among the genes, *OAS1* and *CXCL10* were significantly up-regulated in the obese group (Table S3**)**.

### PPI network construction and hub genes identification for the greenyellow and green modules

**Fig. 4:**
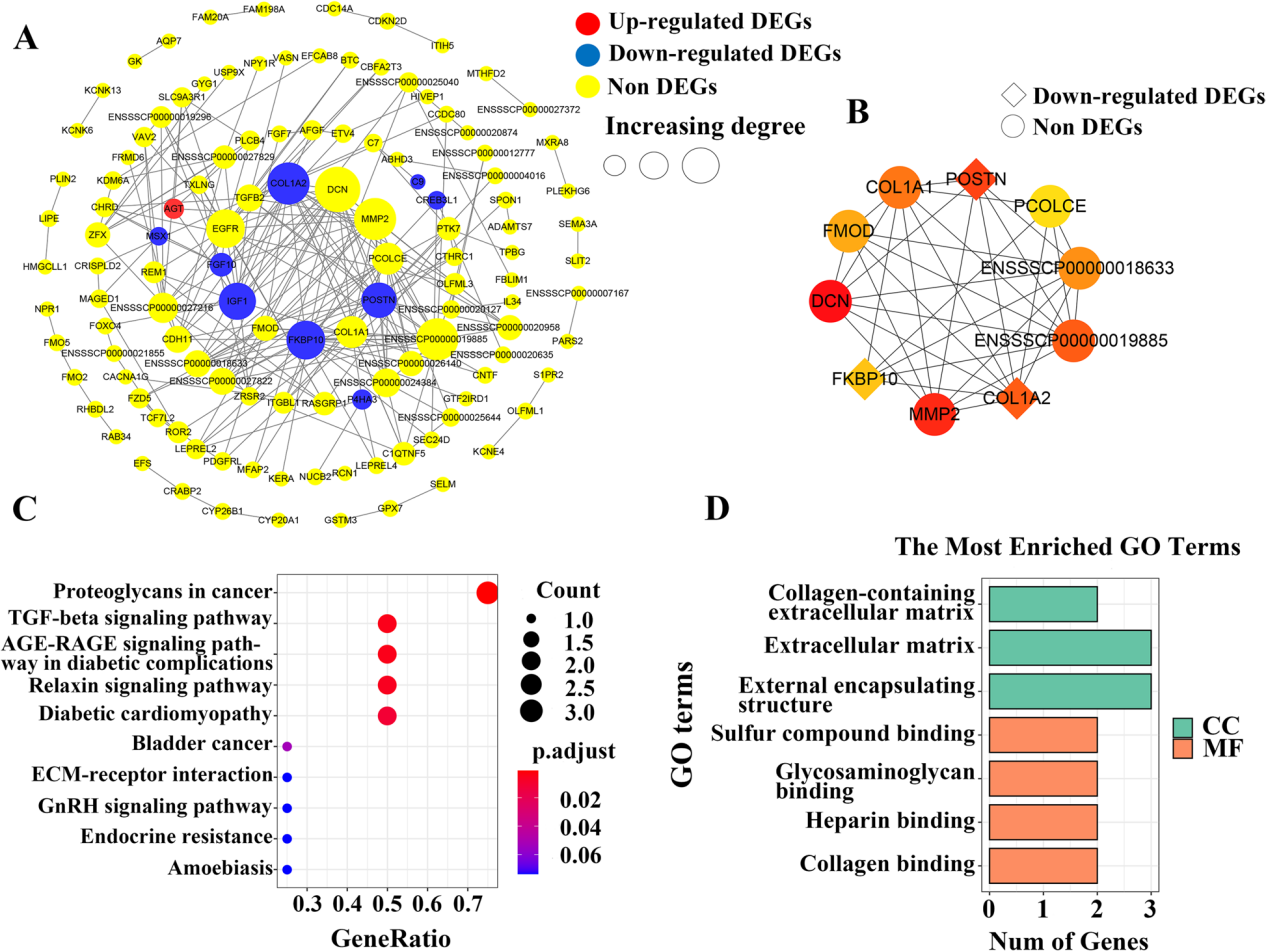
Protein protein interaction (PPI) network for the greenyellow module. **A** The whole PPI network. There were 122 nodes and 238 edges in the network. These nodes (circles) represented genes, and bigger nodes represented genes with more links. Edges (gray lines) between nodes indicated the interaction of genes in the network. Yellow circles represented non DEGs. Red circles represented up-regulated DEGs. Blue circles represented down-regulated DEGs. DEGs (females compared with males). **B** The PPI sub-network. There were 10 nodes and 34 edges in the network. Color represented Maximal Clique Centrality (MCC) score, and the darker the color, the higher MCC score of the node. Diamond nodes represented down-regulated DEGs. DEGs (females compared with males). Functional enrichment analysis for eight hub genes, including KEGG enrichment analysis (**C**) and GO enrichment analysis (**D**). Top 10 terms and top 5 terms ordered by P.adjust for the KEGG and GO enrichment analysis, respectively. P.adjust indicated the degree of enrichment, with smaller P.adjust indicating terms that were more likely to play significantly functional roles

The interactive relationships of all genes in the key module were analyzed by constructing PPI networks. A PPI network, including 122 nodes and 238 edges was constructed for the greenyellow module with a combined score > 0.4 (Fig. 4A). The cytoHubba was used to screen out hub genes in the whole PPI network. According to the Maximal Clique Centrality (MCC) score, the top 10 genes (*DCN*, *MMP2*, *COL1A2*, *FKBP10*, *POSTN*, *COL1A1*, *PCOLCE*, *FMOD*, *ENSSSCG00000019885* and *ENSSSCG00000018633*) were identified as hub genes, and the interactive sub-network, including the 10 hub genes was extracted and established from the whole PPI network (Fig. 4B). Function enrichment analysis showed that the eight genes (except for *ENSSSCG00000019885* and *ENSSSCG00000018633*) were mainly involved in some KEGG pathways, including Proteoglycans in cancer, TGF-beta signaling pathway, AGE-RAGE signaling pathway in diabetic complications, Relaxin signaling pathway, Diabetic cardiomyopathy, Bladder cancer and ECM-receptor interaction (Fig. 4C). The significantly enriched MF terms were Sulfur compound binding, Glycosaminoglycan binding, Heparin binding and Collagen binding. The significantly enriched CC terms were Extracellular matrix, and Collagen-containing extracellular matrix, etc. (Fig. 4D). Three hub genes, *COL1A2*, *POSTN* and *FKBP10* were significantly down-regulated in females compared with males in the obese group (Table S3).

**Fig. 5:**
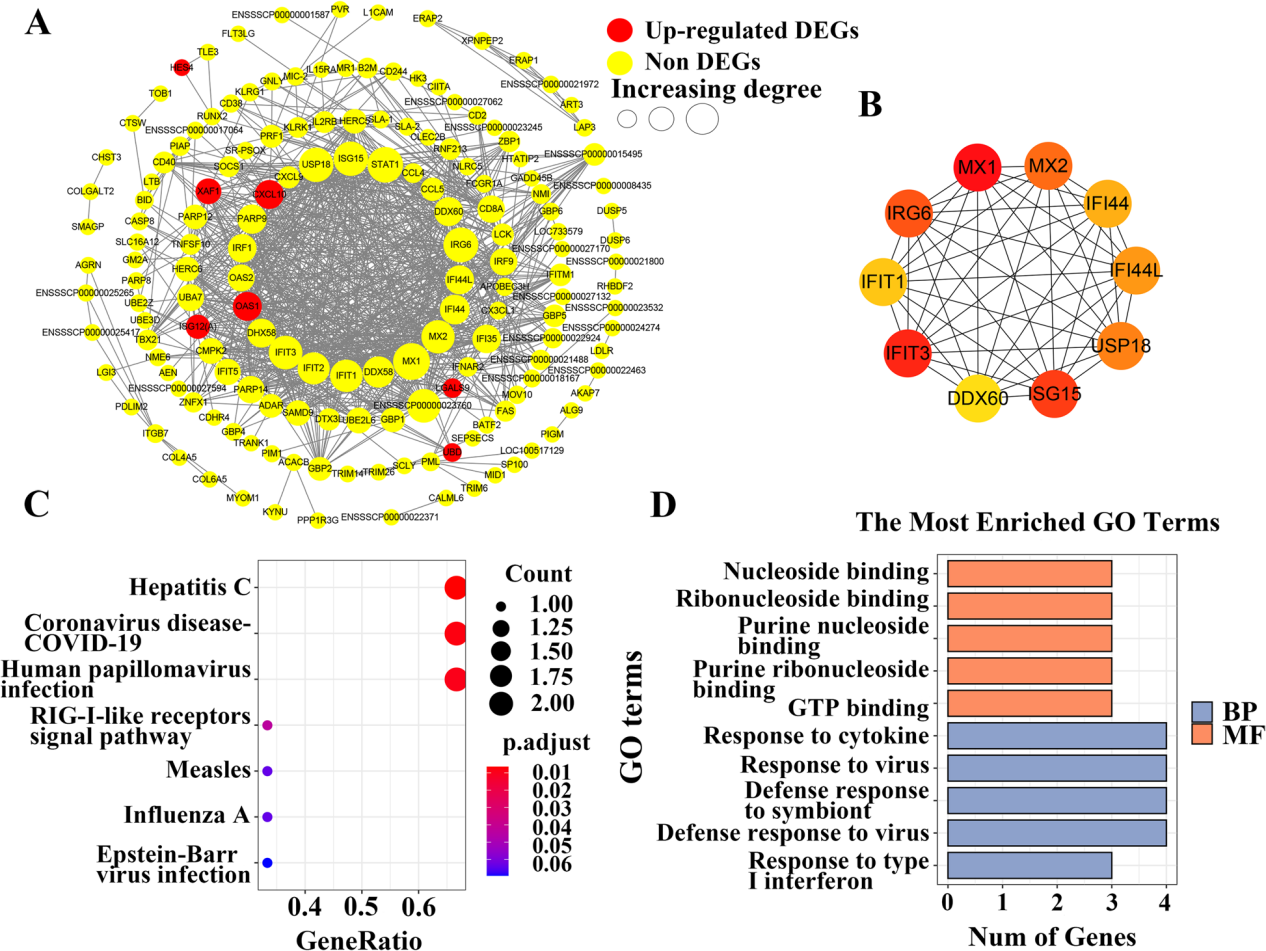
Protein protein interaction (PPI) network for the green module. **A** The whole PPI network. There were 162 nodes and 914 edges in the network. These nodes (circles) represented genes, and bigger nodes represented genes with more links. Edges (gray lines) between nodes indicated the interaction of genes in the network. Yellow circles represented non DEGs. Red circles represented up-regulated DEGs. DEGs (females compared with males). **B** The PPI sub-network. There were 10 nodes and 45 edges in the network. Color represented MCC score, and the darker the color, the higher MCC score of the node. Functional enrichment analysis for 10 hub genes, including KEGG enrichment analysis (**C**) and GO enrichment analysis (**D**). Top 10 terms and top 5 terms ordered by P.adjust for the KEGG and GO enrichment analysis, respectively

A PPI network, including 162 nodes and 914 edges was constructed for the green module with a combined score greater than 0.4 (Fig. 5A). According to the MCC score, 10 hub genes (*MX1*, *MX2*, *IFIT1*, *IFIT3*, *ISG15*, *IRG6*, *IFI44*, *IFI44L*, *USP18* and *DDX60*) were identified and the interactive network was established (Fig. 5B). The 10 hub genes were enriched in some KEGG pathways, including Hepatitis C, Coronavirus disease-COVID-19, Human papillomavirus infection, RIG-I-like receptors signal pathway, Measles, Influenza A and Epstein-Barr virus infection (Fig. 5C). BP analysis showed that these genes were mainly involved in Response to cytokine, Response to virus, Defense response to symbiont, Defense response to virus and Response to type I interferon (Fig. 5D). The enriched MF terms were Nucleoside binding, Ribonucleoside binding, and GTP binding, etc. (Fig. 5D).

## Discussion

### Key sex-specific pathways and genes in the greenyellow module

In our study, a total of 17 coexpression modules were detected using WGCNA method, of which six modules were significantly related to the sexes (*P* < 0.01). Among the significant modules, the greenyellow module was most significantly positively correlated with the male (cor = 0.68, *P* < 9e-06). Functional enrichment analysis showed that the genes in the greenyellow module were mainly involved in Ras signaling pathway, Mitogen-activated protein kinase (MAPK) signaling pathway, Pathways in cancer, Melanoma and Activation of MAPK activity. It is well known that Ras is an important upstream regulator of the MAPK, and the Ras-MAPK signaling pathway can regulate cell proliferation, differentiation, and survival through the kinase cascade [27–29]. Furthermore, four hub genes (*FGF10*, *FGF1*, *EGFR* and *IGF1*) were identified in the greenyellow module by functional enrichment analysis (Fig. 3A). The results showed that *FGF10*, *FGF1* and *EGFR* participated in the Ras signaling pathway and MAPK signaling pathway, and *IGF1* participated in the Ras signaling pathway (Table 1). Insulin-like growth factor (IGF1) can lead to the activation of both MAPK and phosphatidylinositol 3-kinase (PI3K) pathways through Ras [30, 31]. IGF1 is known to stimulate cell proliferation and inhibit apoptosis [32]. A study shows that *IGF1* action is inhibited in the castrated animals, which affects adipocyte proliferation and differentiation [33]. Besides, some studies find that fibroblast growth factor receptor (FGFR) and epidermal growth factor receptor (EGFR) also participate in activating the Ras-MAPK signaling pathway [34, 35]. *FGF1* and *FGF10* belong to the fibroblast growth factor family, which are widely involved in the regulation of cell growth, proliferation, differentiation and regulation of metabolism through *FGFR* [36, 37]. Some studies suggest that *FGF10* stimulates preadipocyte proliferation and differentiation through activating *FGFR2* [38, 39]. As the above, *IGF1*, *FGF1*, *FGF10* and *EGFR* played an important role in activating the Ras-MAPK signaling pathway and promoting adipocyte proliferation and differentiation. Currently, the four genes were not reported in the SATs of pigs of different sexes. Among genes, *FGF10* and *IGF1* were significantly down-regulated in females compared with males in the obese group, while *FGF1* was significantly up-regulated in the obese group. Thus, it could be inferred that *FGF10* and *IGF1* might play key roles in promoting adipocyte proliferation and differentiation in the SATs of boars through the Ras-MAPK signaling pathway.

Besides, eight hub genes, including *COL1A2*, *COL1A1*, *DCN*, *MMP2*, *POSTN*, *FMOD*, *FKBP10* and *PCOLCE* were identified by the PPI network analysis (Fig. 4B). Functional enrichment analysis showed that these genes were significantly enriched in Proteoglycans in cancer, AGE-RAGE signaling pathway in diabetic complications, Relaxin signaling pathway, Extracellular matrix (ECM), ECM-receptor interaction, Collagen binding, and Collagen-containing extracellular matrix, etc. (Fig. 4C, D). The result was very similar to that from the study of Poklukar et al. [33], and their findings showed that the upregulated genes in entire males as compared with immunocastrated males and surgical castrates were significantly enriched in extracellular region/matrix cellular components, ECM receptor interaction and focal adhesion pathways. Some genes responsible for the differences in backfat deposition among the three male sex categories were identified including *COL1A2*, *COL6A3*, *POSTN*, *P4HA3*, *DCN*, *FMOD*, *MMP2* and *MMP27* [33]. In the ECM remodeling, *COL1A2* and *COL1A1* genes involve the synthesis of collagen, which is the major component of ECM [40]. *DCN* (Decorin) gene encodes the ECM protein (DCN), which belongs to the small leucine-rich proteoglycan family. DCN protein can regulate the bioactivities of cell growth factors and participate in ECM assembly [41]. Matrix metalloproteinase 2 (*MMP2*) gene involves ECM degradation [42]. *POSTN* gene is crucial for collagen cross-linking and ECM maintenance [43, 44]. Similarly, *FMOD* gene is required for proper collagen folding and ECM stabilization [45]. *FKBP10* gene is responsible for regulating ECM protein crosslinking and secretion [46]. *PCOLCE* gene can regulate the production of a secreted glycoprotein called procollagen C-proteinase enhancer protein that enhances the activity of procollagen C-proteinases to participate in ECM reconstruction [47, 48]. As above, eight hub genes (*COL1A2*, *COL1A1*, *DCN*, *MMP2*, *POSTN*, *FMOD*, *FKBP10* and *PCOLCE*) played an important role in the ECM remodeling in the SATs of pigs.

Some studies show that ECM remodeling plays many vital roles in ATs. Firstly, it is necessary during the early stage of angiogenesis in ATs [49]. Secondly, it is also associated with the modulation of adipogenesis during adipose tissue expansion [49]. Adipocyte differentiation is regulated by the deposition of collagen (the major component of ECM) [50]. Besides, excess deposition of collagen in obesity can cause AT fibrosis, which leads to AT inflammation by triggering the infiltration of immune cells such as macrophages [51, 52]. A study finds that ECM also participates in activating the Ras-MAPK signaling pathway [53]. Thus, ECM remodeling played an indispensable role in angiogenesis, adipogenesis and adipocyte differentiation of ATs. In this study, three ECM-related genes (*COL1A2*, *POSTN* and *FKBP10*) were significantly down-regulated in females compared with males in the obese group. Jeong et al. measured the expression levels of ECM-related genes in different adipose tissues from bulls, cows and steers of Korean cattle (Hanwoo), and found that the expressions of ECM-related genes in the omental adipose tissue of cows and steers are decreased, and expression levels of most ECM-related genes were generally similar between cows and steers [54]. Poklukar et al. found that castration of male pigs resulted in the downregulation of genes involved in ECM dynamics [33]. The results of these studies were similar to those of this study. As above, it could be speculated that *COL1A2*, *POSTN* and *FKBP10* might play more key roles in promoting angiogenesis and adipogenesis of boars through ECM remodeling in SATs. In summary, two key male-specific pathways (Ras-MAPK signaling pathway and ECM remodeling) and five key male-specific genes (*IGF1*, *FGF10*, *COL1A2*, *POSTN* and *FKBP10*) might play key roles in angiogenesis and adipogenesis in the SATs of male pigs.

### Key sex-specific pathways and genes in the green module

In the current study, the green module was most significantly positively correlated with the female among the significant modules (cor = 0.49, *P* < 0.003). The genes in the green module were mainly enriched in Immune response, Chemokine-mediated signaling pathway, Chemokine activity, Chemokine signaling pathway, Cytokine-cytokine receptor interaction, Cytosolic DNA-sensing pathway, Herpes simplex infection, Measles, and Toll-like receptor signaling pathway, etc. (Table 2). These pathways are closely related to innate immunity and inflammatory response [55–58]. It is well known that Toll-like receptors play an essential role in the innate immune system and inflammatory response [59]. Inflammation is a central component of innate immunity. The inflammatory response involves an increase in the synthesis and secretion of several mediators, including chemokines and cytokines. Chronic inflammation in obesity is directly involved in the etiology of cardiovascular diseases and certain cancer types [60].

Furthermore, eight hub genes, *DDX58*, *OAS1*, *OAS2*, *CXCL9*, *CXCL10*, *CXCL16*, *CCL4* and *CCL5* in the green module were identified by the functional enrichment analysis (Fig. 3B). And 10 hub genes, *MX1*, *MX2*, *IFIT1*, *IFIT3*, *ISG15*, *IRG6*, *IFI44*, *IFI44L*, *USP18* and *DDX60* were identified by the PPI analysis (Fig. 5B). Functional enrichment analysis showed that the 10 hub genes (*MX1*, *MX2*, etc.) were enriched in RIG-I-like receptors (RLRs) signal pathway, Hepatitis C, Immune effector process, Response to virus, Response to type I interferon, and Response to cytokine, etc. (Fig. 5C, D). A study shows that the RLRs play essential roles in the production of type I interferons (IFNs) and proinflammatory cytokines in cell type-specific manners [61]. It has been reported that the *DDX60* gene can promote RLRs receptor signaling [62]. *DDX58* gene belongs to one of the crucial members of the RLRs family, which can promote the production of type I IFN [63, 64]. And then, type I IFN activates kinase-driven signaling to drive the expression of more than 2000 IFN-stimulated genes (ISGs) [65, 66]. As is known to all, Type I IFN plays indispensable roles in immunity and proinflammation via induction of the production of ISGs through activating Janus kinase (JAK)-signal transducer and activator of transcription (STAT) signaling pathway [67]. In this study, the hub genes, including *CXCL9*, *CXCL10*, *CXCL16*, *CCL4* and *CCL5* belong to IFN-induced chemokines [68–70], which participate in the Toll-like receptor signaling pathway. These IFN-induced chemokines might play a vital role in the inflammatory response of SATs from pigs. Some studies show that the 11 hub genes (*OAS1*, *OAS2*, *IFIT1*, *IFIT3*, *ISG15*, *IRG6*, *IFI44*, *IFI44L*, *USP18*, *MX1* and *MX2* were identified in the study) belong to the Type I ISGs, which participate in mediating autoimmune diseases and chronic inflammatory diseases through activating inflammatory responses and innate immunity responses [61, 67, 71].

Currently, the 18 hub genes were not reported in the immunity and inflammation in the SATs of pigs of different sexes. Among 18 genes, *OAS1* and chemokines *CXCL10* were significantly up-regulated in females compared with males in the obese group. The two DEGs might play more key roles in autoimmunity and proinflammation in SATs of the obese female pigs. In summary, some key female-specific pathways and biological processes (Chemokine signaling pathway, Cytokine-cytokine receptor interaction, Toll-like receptor signaling pathway, RLRs signal pathway, Immune response, and Response to type I interferon, etc.) and two key female-specific genes (*CXCL10* and *OAS1*) participating in type I interferon response might play vital roles in innate immunity and proinflammation in the SATs of female pigs.

However, some limitations must be noted in this study. First, the small sample size limited the statistical power to identify the hub genes. Second, molecular biological experiments were required to validate the function of these hub genes in the SATs.

## Conclusions

The systematic associations between SATs and sexes were found, and sex-specific pathways and genes in the SATs of pigs were identified. Males have more abilities in angiogenesis and adipogenesis through activating the Ras-MAPK signaling pathway and ECM remodeling in SATs compared with females. Females have stronger abilities in autoimmunity and proinflammatory via induction of the production of ISGs through activating type I interferon response in SATs compared with males. The identified key sex-specific pathways and genes in SATs from pigs provided some new insights into the molecular mechanism of being involved in fat metabolism and immunoregulation between pigs of different sexes. These findings may be helpful for breeding in the pig industry and obesity treatment in medicine.

## Methods

### Data collection and processing

The transcriptome datasets (GSE61271_normalizeddata.csv.gz) and the phenotypic datasets (GSE61271_series_matrix.txt.gz) were downloaded from the public NCBI GEO database (https://www.ncbi.nlm.nih.gov/geo/query/acc.cgi?acc=GSE61271). The raw sequencing data (100 bp pair-ended fragments, about 30 M reads per sample) were obtained using the Illumina platform. The sequencing samples were collected from the SATs of crossbred F2 pigs (Duroc × Göttingen minipig). Göttingen minipig is genetically susceptible to obesity and shares a variety of metabolic diseases with humans [72]. According to the descriptions of the original paper [8], the 36 F2 pigs (17 females and 19 males) were produced at the research farm, the University of Copenhagen Tåstrup, Denmark. Basing on the selection index theory, Kogelman et al. created the Obesity Index (OI) to represent the degree of obesity in each pig. According to OI, 36 pigs were categorized into three groups: 12 low OI (Lean, L), 12 intermediate OI (Intermediate, I), and 12 high OI (Obese, O). Among the selected pigs, there was a large difference in age at slaughter (L: 309 days, I: 234 days, O: 218 days), as they were slaughtered at approximately 100 kg.

In order to balance the sample number of male and female pigs, two samples of males (GSM1501206 and GSM1501208) in the lean group were randomly eliminated. A total of 34 samples (17 females and 17 males) were selected for this study. The samples with different obesity levels in the three groups were evenly distributed in the two sex groups. Details about samples were shown in Table 3 and Table S1.

**Table 3:** The sample information of 34 pigs

Sex	Total	Lean	Intermediate	Obese
Females	17	5	6	6
Males	17	5	6	6

According to Obesity Index (OI), 34 pigs (17 females and 17 males) were divided into three groups: the Lean, Intermediate and Obese groups, which represented different obesity levels of pigs in each group

### Differential expression genes analysis

The transcriptome datasets, including 5000 genes were used to construct the expression matrix. Differential expression analysis of the females and males in three groups (Lean, Intermediate and Obese groups) was performed separately using the limma package [73]. In the study, genes with |log2FC|> 1 and FDR < 0.1 were referred to as the differentially expressed genes (DEGs). The DEGs were visualized as a volcano plot using the R package ggplot2, while as a heatmap plot using the R function pheatmap.

### WGCNA

WGCNA was used to construct the gene coexpression network, and identify the coexpression gene modules. The WGCNA package (version 1.13) based on R was used to perform WGCNA [15]. First, the expression matrix was converted into an adjacency matrix, and an unsupervised coexpression relationship was constructed based on the adjacency matrix using Pearson correlation coefficients for gene pairs. The correlation adjacency matrix was strengthened by power β (soft threshold), and the power parameter was selected based on the scale-free topology criterion.

Second, the adjacency matrix was transformed into a topology matrix. TOM was used to measure the correlation of gene pairs. According to 1-TOM, average linkage hierarchical clustering was performed to classify genes with coherent expression profiles into gene modules. The dynamic cutting algorithm was used to identify gene modules from the system cluster tree. Module eigengene (ME) was defined as the first principal component and was the representative of module genes. Module membership (MM) was defined as the correlation between ME and gene module. Gene significance (GS) was indexed by log10 transformation of the *P*-value of the T-test. GS of 0 indicates that the gene was not significant with regard to the biological question of interest. The GS could take on positive or negative values. Module significance (MS) was defined as the average of GS for all the genes in the module. A more detailed description of WGCNA was presented in an original article [13].

Finally, the statistical significance of the relationship between modules and sexes was analyzed by calculating the Pearson correlation coefficient. For studying the genes in the module correlating with sexes, modules with *p* values < 0.01 were selected as significant modules in this study. And then, the module with the significant positive correlation (cor > 0) with males and females among all the significant modules was selected as the key module for further analysis, respectively.

### PPI network construction and analysis

The interactive relationships among genes encoding proteins in the key gene coexpression module were analyzed by constructing a PPI network. The interactive information among genes encoding proteins was retrieved from the Search Tool for the Retrieval of Interacting Genes (STRING) database (version 11.5, https://string-db.org/). The gene pairs with a combined score ≥ the medium confidence of 0.4 were used to construct the PPI network. The Cytoscape (v3.8.0) software was used to construct and visualize the interactive relationships among genes in the whole PPI network [74].

### Functional enrichment analysis

GO and KEGG pathway terms of all genes in the key module were analyzed using the online DAVID database (version 6.80, https://david.ncifcrf.gov/) [75]. The cut-off criterion was set at *P*-value < 0.05. Cytoscape (v3.8.0) software was used to construct and visualize the interactive relationships between genes and functional enrichment terms in the whole network. Functional enrichment analysis for hub genes in the PPI sub-network was implemented using the R-package clusterProfiler [75, 76]. The cut-off criterion of KEGG was set at *P*-value < 0.1, and the cut-off criterion of GO was set at *P*-value < 0.01 and q-value < 0.05. GO annotation result includes three main bodies: biological process (BP), molecular function (MF) and cellular component (CC).

### Hub genes identification

Hub genes in the whole PPI network from the key modules were identified by the cytoHubba algorithm in the Cytoscape software, and the criterion for selecting hub genes was that the top 10 nodes ranked by Maximal Clique Centrality (MCC) [77]. Key genes in key modules were identified using the functional enrichment network analysis. The selection criterion of key genes in the module was that the gene was at least involved in four KEGG/GO terms.

## Supplementary Information

**Additional file 1:** **Table S1.**Grouping information of 34 samples.

**Additional file 2:** **Table S2.**The number of genes in each of the 17 modules.

**Additional file 3:** **Table S3.** The results of differentially expressed genes (DEGs) analysis for the obese group.

## Data Availability

The transcriptome datasets (GSE61271_normalizeddata.csv.gz) and the phenotypic datasets (GSE61271_series_matrix.txt.gz) analyzed during the current study are available in the public NCBI GEO database (https://www.ncbi.nlm.nih.gov/geo/query/acc.cgi?acc=GSE61271) [8].

## Abbreviations

AT: Adipose tissue
VAT: Visceral AT
SAT: Subcutaneous AT
ASAT: Abdominal SAT
GEO: Gene Expression Omnibus
DEGs: Differential expression genes
WGCNA: Weighted gene coexpression network analysis
TOM: Topological overlap measure
1-TOM: TOM-based dissimilarity
ME: Module eigengene
GS: Gene significance
MM: Module membership
MS: Module significance
DAVID: Database for Annotation, Visualization and Integrated Discovery
STRING: Search Tool for the Retrieval of Interacting Genes
PPI: Protein and protein interaction
MCC: Maximal clique centrality
ECM: Extracellular matrix
MAPK: Mitogen-activated protein kinase
JAK-STAT: Janus kinase-signal transducer and activator of transcription
IFN: Interferon
ISG: IFN-stimulated gene
RLR: RIG-I-like receptor
BP: Biological process
MF: Molecular function
CC: Cellular component
