# Supplementary material for: Identification of key sex-specific pathways and genes in the subcutaneous adipose tissue from pigs using WGCNA method
Source: BMC Genom Data. 2022 May 10;23:35. doi: 10.1186/s12863-022-01054-w (PMC9086418; doi:10.1186/s12863-022-01054-w)
Supplement: Supplementary file 1 — Additional file 1: Table S1.Grouping information of 34 samples. [file 12863_2022_1054_MOESM1_ESM.docx]

| **Samples** | **Sex** | **OI** | **Abbr.** | **Samples** | **Sex** | **OI** | **Abbr.** |
| --- | --- | --- | --- | --- | --- | --- | --- |
| GSM1501205 | Female | Lean | F_L_1 | GSM1501209 | Male | Lean | M_L_1 |
| GSM1501207 | Female | Lean | F_L_2 | GSM1501211 | Male | Lean | M_L_2 |
| GSM1501210 | Female | Lean | F_L_3 | GSM1501213 | Male | Lean | M_L_3 |
| GSM1501212 | Female | Lean | F_L_4 | GSM1501214 | Male | Lean | M_L_4 |
| GSM1501216 | Female | Lean | F_L_5 | GSM1501215 | Male | Lean | M_L_5 |
| GSM1501217 | Female | Intermediate | F_I_1 | GSM1501218 | Male | Intermediate | M_I_1 |
| GSM1501219 | Female | Intermediate | F_I_2 | GSM1501222 | Male | Intermediate | M_I_2 |
| GSM1501220 | Female | Intermediate | F_I_3 | GSM1501223 | Male | Intermediate | M_I_3 |
| GSM1501221 | Female | Intermediate | F_I_4 | GSM1501225 | Male | Intermediate | M_I_4 |
| GSM1501224 | Female | Intermediate | F_I_5 | GSM1501227 | Male | Intermediate | M_I_5 |
| GSM1501226 | Female | Intermediate | F_I_6 | GSM1501228 | Male | Intermediate | M_I_6 |
| GSM1501229 | Female | Obese | F_O_1 | GSM1501232 | Male | Obese | M_O_1 |
| GSM1501230 | Female | Obese | F_O_2 | GSM1501233 | Male | Obese | M_O_2 |
| GSM1501231 | Female | Obese | F_O_3 | GSM1501234 | Male | Obese | M_O_3 |
| GSM1501235 | Female | Obese | F_O_4 | GSM1501236 | Male | Obese | M_O_4 |
| GSM1501237 | Female | Obese | F_O_5 | GSM1501239 | Male | Obese | M_O_5 |
| GSM1501238 | Female | Obese | F_O_6 | GSM1501240 | Male | Obese | M_O_6 |

**Table S1** Grouping information of 34 samples
