# Supplementary material for: Identification of key sex-specific pathways and genes in the subcutaneous adipose tissue from pigs using WGCNA method
Source: BMC Genom Data. 2022 May 10;23:35. doi: 10.1186/s12863-022-01054-w (PMC9086418; doi:10.1186/s12863-022-01054-w)
Supplement: Supplementary file 2 — Additional file 2: Table S2.The number of genes in each of the 17 modules. [file 12863_2022_1054_MOESM2_ESM.docx]

**Table S2** The number of genes in each of the 17 modules

| **Module color** | Black | Blue | Brown | Cyan | Green | Green  yellow | Grey | Midnignt  blue | Lightcyan |
| --- | --- | --- | --- | --- | --- | --- | --- | --- | --- |
| **Gene number** | 241 | 677 | 491 | 161 | 258 | 205 | 268 | 155 | 137 |
| **Module color** | Magenta | Pink | Purple | Red | Tan | Salmon | Yellow | Turquoise |  |
| **Gene number** | 216 | 237 | 205 | 255 | 168 | 164 | 304 | 855 |  |
