# Supplementary material for: Identification of key sex-specific pathways and genes in the subcutaneous adipose tissue from pigs using WGCNA method
Source: BMC Genom Data. 2022 May 10;23:35. doi: 10.1186/s12863-022-01054-w (PMC9086418; doi:10.1186/s12863-022-01054-w)
Supplement: Supplementary file 3 — Additional file 3: Table S3. The results of differentially expressed genes (DEGs) analysis for the obese group. [file 12863_2022_1054_MOESM3_ESM.docx]

**Table S3** The results of differentially expressed genes (DEGs) analysis for the obese group

| **Ensembl ID** | **[Gene Symbol](http://www.baidu.com/link?url=bvat91kSduTlNrhQZYzvndRLo8atI8VTHLNwvO2NAROI067U7PU9FktKu0zcqD7g)s** | **Log2FC** | **P.Value** | **FDR** | **Change** |
| --- | --- | --- | --- | --- | --- |
| ENSSSCG00000016878 | FGF10* | -2.492791667 | 2.16E-07 | 0.001081851 | DOWN |
| ENSSSCG00000012730 | AFF2 | -2.21867 | 4.43E-07 | 0.001108313 | DOWN |
| ENSSSCG00000013393 | SPON1 | -1.71165 | 7.50E-06 | 0.012496488 | DOWN |
| ENSSSCG00000010654 | ATRNL1 | -1.485558333 | 1.78E-05 | 0.015224956 | DOWN |
| ENSSSCG00000016983 | STC2 | -2.216765 | 1.83E-05 | 0.015224956 | DOWN |
| ENSSSCG00000000857 | IGF1* | -1.345850703 | 2.15E-05 | 0.015354952 | DOWN |
| ENSSSCG00000016715 | OSBPL3 | -1.523725 | 2.96E-05 | 0.018523484 | DOWN |
| ENSSSCG00000016856 | C9 | -1.296688333 | 4.16E-05 | 0.020854258 | DOWN |
| ENSSSCG00000010437 | PAPSS2 | -1.244841667 | 4.41E-05 | 0.020854258 | DOWN |
| ENSSSCG00000029781 | SELENOM | -1.15597 | 4.79E-05 | 0.020854258 | DOWN |
| ENSSSCG00000010698 | FGFR2 | -1.473698333 | 5.42E-05 | 0.020854258 | DOWN |
| ENSSSCG00000008422 |  | -1.1507 | 6.10E-05 | 0.021635382 | DOWN |
| ENSSSCG00000011998 | VGLL3 | -1.334896667 | 6.92E-05 | 0.021635382 | DOWN |
| ENSSSCG00000013263 | CREB3L1 | -1.632246667 | 7.53E-05 | 0.022160974 | DOWN |
| ENSSSCG00000012156 | CDKL5 | -1.290241667 | 9.47E-05 | 0.026015751 | DOWN |
| ENSSSCG00000008841 | PDGFRA | -1.25396 | 0.000109241 | 0.026015751 | DOWN |
| ENSSSCG00000008555 | CGREF1 | -1.26224 | 0.000129858 | 0.026015751 | DOWN |
| ENSSSCG00000005186 | TTC39B | -1.17771 | 0.000136763 | 0.026015751 | DOWN |
| ENSSSCG00000000927 | TMTC3 | -1.20329896 | 0.000143144 | 0.026015751 | DOWN |
| ENSSSCG00000015410 | PHTF2 | -1.132278333 | 0.000145012 | 0.026015751 | DOWN |
| ENSSSCG00000006729 | TENT5C | -1.168093333 | 0.000150989 | 0.026015751 | DOWN |
| ENSSSCG00000010746 | ADAM12 | -1.668408333 | 0.000160197 | 0.026015751 | DOWN |
| ENSSSCG00000007170 | CPXM1 | -1.527631667 | 0.000164294 | 0.026015751 | DOWN |
| ENSSSCG00000000164 | CRY1 | -1.215936652 | 0.000168767 | 0.026015751 | DOWN |
| ENSSSCG00000021015 |  | -1.608398333 | 0.00017306 | 0.026015751 | DOWN |
| ENSSSCG00000008727 | MSX1 | -1.257501667 | 0.000181481 | 0.026015751 | DOWN |
| ENSSSCG00000017579 | COL1A1 | -1.584833333 | 0.00018211 | 0.026015751 | DOWN |
| ENSSSCG00000029074 |  | -1.55618 | 0.000255353 | 0.032737508 | DOWN |
| ENSSSCG00000021943 |  | -1.526245 | 0.000268153 | 0.032914589 | DOWN |
| ENSSSCG00000014203 | MCC | -1.124043333 | 0.000277751 | 0.032914589 | DOWN |
| ENSSSCG00000027593 |  | -1.566826667 | 0.000296411 | 0.033683123 | DOWN |
| ENSSSCG00000017581 |  | -1.534653333 | 0.000323805 | 0.03597829 | DOWN |
| ENSSSCG00000025831 | P311 | -1.588918333 | 0.000337997 | 0.036011764 | DOWN |
| ENSSSCG00000023444 |  | -1.430373333 | 0.000346804 | 0.036125379 | DOWN |
| ENSSSCG00000015326 | COL1A2* | -1.445518333 | 0.00040745 | 0.038853146 | DOWN |
| ENSSSCG00000006338 | DDR2 | -1.353818333 | 0.000411843 | 0.038853146 | DOWN |
| ENSSSCG00000004907 | CCBE1 | -1.348958333 | 0.000443534 | 0.039963939 | DOWN |
| ENSSSCG00000014088 | IQGAP2 | -1.243408333 | 0.000497316 | 0.041337364 | DOWN |
| ENSSSCG00000001849 | ANPEP | -1.31991795 | 0.000517853 | 0.041337364 | DOWN |
| ENSSSCG00000004650 | FGF7 | -1.24121 | 0.000570679 | 0.041435247 | DOWN |
| ENSSSCG00000022000 |  | -1.424305 | 0.000571806 | 0.041435247 | DOWN |
| ENSSSCG00000021549 | DTWD2 | -1.157418333 | 0.000592635 | 0.042234582 | DOWN |
| ENSSSCG00000012527 | TCEAL9 | -1.122673333 | 0.000754558 | 0.046197783 | DOWN |
| ENSSSCG00000006842 |  | -1.332678333 | 0.000763924 | 0.046197783 | DOWN |
| ENSSSCG00000005992 | SHAS2 | -1.86728 | 0.000778941 | 0.046197783 | DOWN |
| ENSSSCG00000016074 | ANKRD44 | -1.114921667 | 0.000802371 | 0.046649488 | DOWN |
| ENSSSCG00000021206 | IL1RAP | -1.58889 | 0.000828805 | 0.047632462 | DOWN |
| ENSSSCG00000030325 | C1QTNF6 | -1.54298 | 0.000885907 | 0.049692811 | DOWN |
| ENSSSCG00000006971 |  | -1.52772 | 0.000894471 | 0.049692811 | DOWN |
| ENSSSCG00000022673 |  | -1.211331667 | 0.001002919 | 0.052541659 | DOWN |
| ENSSSCG00000020725 | ERBB3 | -1.40351 | 0.00105834 | 0.052541659 | DOWN |
| ENSSSCG00000003366 | CHD5 | -1.881293333 | 0.001267052 | 0.057593257 | DOWN |
| ENSSSCG00000007903 | EMP2 | -1.28053 | 0.001393584 | 0.059546875 | DOWN |
| ENSSSCG00000025427 |  | -1.221145 | 0.001489216 | 0.059546875 | DOWN |
| ENSSSCG00000004668 | SLC30A4 | -1.148078333 | 0.001489652 | 0.059546875 | DOWN |
| ENSSSCG00000021732 | CRYBG3 | -1.170526667 | 0.001507776 | 0.059546875 | DOWN |
| ENSSSCG00000015814 | TACC1 | -1.492405 | 0.001510624 | 0.059546875 | DOWN |
| ENSSSCG00000016194 | USP37 | -1.227775 | 0.00151798 | 0.059546875 | DOWN |
| ENSSSCG00000024812 |  | -1.23896 | 0.001538775 | 0.059546875 | DOWN |
| ENSSSCG00000016031 | CALCRL | -1.201645 | 0.001698674 | 0.060789429 | DOWN |
| ENSSSCG00000028487 | DBNDD2 | -1.190773333 | 0.00172714 | 0.060789429 | DOWN |
| ENSSSCG00000027367 |  | -1.248265 | 0.001756742 | 0.060789429 | DOWN |
| ENSSSCG00000003764 | PTGFR | -1.368775 | 0.001827195 | 0.061729551 | DOWN |
| ENSSSCG00000020912 | HECTD2 | -1.225118333 | 0.001845159 | 0.061918091 | DOWN |
| ENSSSCG00000014087 |  | -1.34742 | 0.002089585 | 0.065929852 | DOWN |
| ENSSSCG00000015589 | VASH2 | -1.206781667 | 0.002354146 | 0.070483404 | DOWN |
| ENSSSCG00000014840 | P4HA3 | -2.223138333 | 0.002405873 | 0.071200879 | DOWN |
| ENSSSCG00000013909 | CRLF1 | -1.819073333 | 0.00249336 | 0.071238859 | DOWN |
| ENSSSCG00000006342 | UHMK1 | -1.193505 | 0.002661121 | 0.071617609 | DOWN |
| ENSSSCG00000011292 | ZNF662 | -1.170126667 | 0.002668419 | 0.071617609 | DOWN |
| ENSSSCG00000014012 | GFPT2 | -1.272405 | 0.00271034 | 0.071617609 | DOWN |
| ENSSSCG00000003811 | ROR1 | -1.121553333 | 0.002913165 | 0.07393821 | DOWN |
| ENSSSCG00000016396 | TNFAIP6 | -1.221063333 | 0.002986452 | 0.074728717 | DOWN |
| ENSSSCG00000005941 | KHDRBS3 | -1.420231667 | 0.00301158 | 0.074914921 | DOWN |
| ENSSSCG00000005710 | LAMC3 | -1.609608333 | 0.003029663 | 0.074991669 | DOWN |
| ENSSSCG00000009361 | POSTN* | -1.976485 | 0.003633196 | 0.077632403 | DOWN |
| ENSSSCG00000017422 | FKBP10* | -1.116251667 | 0.003852138 | 0.078798738 | DOWN |
| ENSSSCG00000007872 | XYLT1 | -1.156143333 | 0.004052581 | 0.079775212 | DOWN |
| ENSSSCG00000015766 | WDR17 | -1.22517 | 0.004402386 | 0.082675599 | DOWN |
| ENSSSCG00000015796 | PDLIM3 | -1.598785 | 0.004453371 | 0.082675599 | DOWN |
| ENSSSCG00000026445 | CGNL1 | -1.251416667 | 0.004607137 | 0.083766122 | DOWN |
| ENSSSCG00000024685 | GRIA2 | -1.179868333 | 0.004761069 | 0.08471653 | DOWN |
| ENSSSCG00000021440 | GPSM2 | -1.350421667 | 0.005529622 | 0.092778887 | DOWN |
| ENSSSCG00000026686 | PDZD9 | -1.26115 | 0.005612706 | 0.09331327 | DOWN |
| ENSSSCG00000008177 | REV1 | -1.208255 | 0.00638384 | 0.096456772 | DOWN |
| ENSSSCG00000011666 | CLSTN2 | -1.394563333 | 0.006462604 | 0.096456772 | DOWN |
| ENSSSCG00000006340 | UAP1 | -1.141713333 | 0.006622895 | 0.097219673 | DOWN |
| ENSSSCG00000028411 | MC5R | 2.24631 | 1.40E-05 | 0.015224956 | UP |
| ENSSSCG00000017105 | UBE2QL1 | 1.711855 | 5.34E-05 | 0.020854258 | UP |
| ENSSSCG00000001808 | CPEB1 | 1.638893643 | 6.49E-05 | 0.021635382 | UP |
| ENSSSCG00000011721 | P2RY1 | 1.54201 | 0.000107749 | 0.026015751 | UP |
| ENSSSCG00000016892 | FST | 1.651606667 | 0.000144402 | 0.026015751 | UP |
| ENSSSCG00000003744 | MOCOS | 1.29132 | 0.000162689 | 0.026015751 | UP |
| ENSSSCG00000007146 | SIGLEC1 | 1.641023333 | 0.000198247 | 0.026790179 | UP |
| ENSSSCG00000006495 | SEMA4A | 1.540358333 | 0.000223308 | 0.029382628 | UP |
| ENSSSCG00000000759 | WNT5B | 1.287399273 | 0.000283065 | 0.032914589 | UP |
| ENSSSCG00000015089 | JAML | 1.456885 | 0.000338511 | 0.036011764 | UP |
| ENSSSCG00000015022 | LAYN | 1.340053333 | 0.000364709 | 0.037044557 | UP |
| ENSSSCG00000015512 | PAPPA2 | 1.606485 | 0.000370446 | 0.037044557 | UP |
| ENSSSCG00000016215 | LOC100158003 | 1.420655 | 0.000388211 | 0.038059895 | UP |
| ENSSSCG00000003230 | CLDND2 | 1.305448333 | 0.000447596 | 0.039963939 | UP |
| ENSSSCG00000002893 | CD22 | 1.34389 | 0.000455975 | 0.039997764 | UP |
| ENSSSCG00000014834 | UCP3 | 1.494856667 | 0.000467387 | 0.040291981 | UP |
| ENSSSCG00000013583 | CAMSAP3 | 1.27205 | 0.000516624 | 0.041337364 | UP |
| ENSSSCG00000024430 | HES4 | 1.597481667 | 0.000520851 | 0.041337364 | UP |
| ENSSSCG00000010101 | P2RX6 | 1.17634 | 0.000599731 | 0.042234582 | UP |
| ENSSSCG00000010184 | AGT | 1.367151667 | 0.000611621 | 0.042473682 | UP |
| ENSSSCG00000006245 | SDR16C5 | 1.326543333 | 0.000706211 | 0.045269908 | UP |
| ENSSSCG00000024954 | FGF1* | 1.870253333 | 0.000727958 | 0.045497393 | UP |
| ENSSSCG00000017103 | SRD5A1 | 1.143393333 | 0.000785362 | 0.046197783 | UP |
| ENSSSCG00000001551 | CLPSL2 | 1.469678227 | 0.001039222 | 0.052541659 | UP |
| ENSSSCG00000003975 | KCNQ4 | 1.392891667 | 0.001469678 | 0.059546875 | UP |
| ENSSSCG00000016530 | TMEM140 | 1.65895 | 0.001787209 | 0.060789429 | UP |
| ENSSSCG00000016473 | EPHB6 | 1.327741667 | 0.002096569 | 0.065929852 | UP |
| ENSSSCG00000000810 | AMIGO2 | 1.132747785 | 0.00247239 | 0.071204667 | UP |
| ENSSSCG00000016403 | VIPR2 | 1.323495 | 0.002735793 | 0.071617609 | UP |
| ENSSSCG00000021241 | ADAMTS13 | 1.833476667 | 0.003056239 | 0.075074639 | UP |
| ENSSSCG00000007797 | ITGAL | 1.194576667 | 0.003063045 | 0.075074639 | UP |
| ENSSSCG00000011610 | NUP210 | 1.248848333 | 0.003378573 | 0.077577225 | UP |
| ENSSSCG00000008965 | BTC | 1.171758333 | 0.003397882 | 0.077577225 | UP |
| ENSSSCG00000010478 | FFAR4 | 1.281898333 | 0.003431703 | 0.077632403 | UP |
| ENSSSCG00000009889 | ALDH2 | 1.128115 | 0.003553043 | 0.077632403 | UP |
| ENSSSCG00000021391 |  | 1.356841667 | 0.003597047 | 0.077632403 | UP |
| ENSSSCG00000000363 | GDF11 | 1.231892563 | 0.003615672 | 0.077632403 | UP |
| ENSSSCG00000002818 | PLLP | 1.349068333 | 0.003624976 | 0.077632403 | UP |
| ENSSSCG00000002471 | ISG12(A) | 1.57575687 | 0.003868955 | 0.078798738 | UP |
| ENSSSCG00000012135 | VEGFD | 1.201996667 | 0.004014322 | 0.079775212 | UP |
| ENSSSCG00000008977 | CXCL10* | 3.355205 | 0.00429507 | 0.08165532 | UP |
| ENSSSCG00000009882 | OAS1* | 1.585251667 | 0.004501876 | 0.082675599 | UP |
| ENSSSCG00000025503 | ADGRG5 | 1.254526667 | 0.005844383 | 0.09331327 | UP |
| ENSSSCG00000017887 | XAF1 | 1.79264 | 0.005911236 | 0.093829145 | UP |
| ENSSSCG00000017754 | LGALS9 | 1.522561667 | 0.006397561 | 0.096456772 | UP |
| ENSSSCG00000006328 | RXRG | 1.52802 | 0.006801837 | 0.098292437 | UP |
| ENSSSCG00000001252 | UBD | 1.906148455 | 0.00694763 | 0.099536248 | UP |

Genes marked with * represent key genes. FDR is adjusted p-value using the false discovery rate (FDR) method.
